# Endothelial Dll4 overexpression reduces vascular response and inhibits tumor growth and metastasization in vivo

**DOI:** 10.1186/s12885-017-3171-2

**Published:** 2017-03-14

**Authors:** Alexandre Trindade, Dusan Djokovic, Joana Gigante, Liliana Mendonça, António Duarte

**Affiliations:** 1 Centro Interdisciplinar de Investigação em Sanidade Animal (CIISA), Faculdade de Medicina Veterinária, University of Lisbon, Lisbon, Portugal; 2 Faculdade de Ciências Médicas, Nova Medical School, Nova University of Lisbon, Lisbon, Portugal; 3 Serviço de Obstetrícia e Ginecologia, Centro Hospitalar de Lisboa Ocidental, Hospital de São Francisco Xavier, Lisbon, Portugal

**Keywords:** Angiogenesis, Dll4, Notch, Overexpression, Tumor, Metastasis, Drug-delivery

## Abstract

**Background:**

The inhibition of Delta-like 4 (Dll4)/Notch signaling has been shown to result in excessive, nonfunctional vessel proliferation and significant tumor growth suppression. However, safety concerns emerged with the identification of side effects resulting from chronic Dll4/Notch blockade. Alternatively, we explored the endothelial Dll4 overexpression using different mouse tumor models.

**Methods:**

We used a transgenic mouse model of endothelial-specific Dll4 overexpression, previously produced. Growth kinetics and vascular histopathology of several types of solid tumors was evaluated, namely Lewis Lung Carcinoma xenografts, chemically-induced skin papillomas and RIP1-Tag2 insulinomas.

**Results:**

We found that increased Dll4/Notch signaling reduces tumor growth by reducing vascular endothelial growth factor (VEGF)-induced endothelial proliferation, tumor vessel density and overall tumor blood supply. In addition, Dll4 overexpression consistently improved tumor vascular maturation and functionality, as indicated by increased vessel calibers, enhanced mural cell recruitment and increased network perfusion. Importantly, the tumor vessel normalization is not more effective than restricted vessel proliferation, but was found to prevent metastasis formation and allow for increased delivery to the tumor of concomitant chemotherapy, improving its efficacy.

**Conclusions:**

By reducing endothelial sensitivity to VEGF, these results imply that Dll4/Notch stimulation in tumor microenvironment could be beneficial to solid cancer patient treatment by reducing primary tumor size, improving tumor drug delivery and reducing metastization. Endothelial specific Dll4 overexpression thus appears as a promising anti-angiogenic modality that might improve cancer control.

## Background

Current angiogenic inhibitors targeting VEGF signaling show therapeutic efficacy in many aggressive tumors, but fail to provide enduring clinical benefit in most cases [1–7].

In addition, the VEGF inhibitors with documented anti-tumor efficacy have been found in mouse models to be prone to elicit tumor adaptation and progression to stages of greater malignancy, with heightened invasiveness and in some cases increased lymphatic and distant metastasis [8]. Therefore, there is a need to validate novel therapeutic targets alongside VEGF signaling inhibition. In this context, Dll4/Notch signaling appears as a promising candidate.

Acting downstream of VEGF signaling, Dll4/Notch signaling essentially contributes to proper vascular remodeling during embryonic vascular development [9–11]. The endothelial ligand Dll4 interacts with Notch 1 receptors of adjacent endothelial cells, triggering γ-secretase proteolytic cleavage of Notch intracellular domain (NICD), which subsequently translocates to the nucleus as a complex with the recombination signal binding protein Jκ (RBP-Jκ) and activates effector genes including the members of the Hes and Hey families of basic helix-loop-helix transcription factors [12] and EphrinB2 [13]. In the postnatal period Dll4 has a low level of expression in quiescent blood vessels of normal tissue [14]. However, its up-regulation is a hallmark of proliferating tumor vessels in both preclinical murine models [10, 15–18] and different human malignancies [14, 19–21]. Besides, Dll4 expression was identified in malignant cells of different types [22], and the ligand was shown to have an impact on colon cancer stem cell frequency by suppressing apoptosis of tumor cells [23, 24]. Nevertheless, vascular endothelium represents the most prominent and constant site of Dll4 expression within tumors.

Targeted *Dll4* allele deletion, local overexpression of Dll4/Notch-blockers, systemic application of soluble Dll4/Notch-inhibitors and DNA vaccination were found to result in a significant suppression of tumor growth in numerous preclinical models, including malignancies resistant to VEGF inhibitors [15–17, 25–28]. Tumor growth retardation due to Dll4/Notch inhibition is associated with an apparently paradoxical increase of endothelial proliferation, migration and subsequent tumor vessel density, but also excessive branching with defective lumenization and impaired mural cell recruitment, both leading to non-functional vessel formation and subsequent tumor starvation. Nevertheless, the normalization capacity of these vascular aberrations remains undetermined as well as the persistence of beneficial effects attributed to the Dll4/Notch inhibition. Additionally, poor blood perfusion raises concerns that therapeutic Dll4/Notch inhibition may reduce the effectiveness of concomitant chemotherapy while hypoxia can contribute to more malignant cell selection [29]. Besides, chronic Dll4 blockade was found to disrupt normal organ vascular homeostasis and induce vascular tumor formation [15, 30, 31]. Thus, in certain contexts, the putative side effects of blocking Dll4/Notch pathway may come to limit its clinical usefulness.

The vessel defects induced by the Dll4/Notch inhibition closely resemble vascular abnormalities commonly observed in human malignancies; so increased Dll4 expression in tumor vasculature, that reduces endothelial sensitivity to VEGF [32], may be considered a host defense mechanism serving to restrict tumor vascularization and malignant cell access to the bloodstream such as Angiopoietin-2 does in the case of vascular cooption [33]. Thus, amplification of Dll4/Notch signals appears as a rational therapeutic option to be tested. Virus-transduced malignant cells that over-express Dll4 activate Notch signaling in co-cultured endothelial cells and restrict VEGF-induced endothelial cell growth [17, 26]. When expressed in tumor cells, Dll4 also functions as a negative regulator of tumor angiogenesis; however, there is no consistent information on the effects of Dll4 over-expression on tumor kinetics. While it was reported to act as a negative driver of tumor expansion in tested malignant cell lines grafted in mice [17, 26, 34], Dll4 expression in transduced human glioblastoma, prostate and gastric cancer cell xenografts was associated with promoted tumor growth, to some extent, due to a reduction of tumor hypoxia and apoptosis or increased secretion of matrix metalloproteinase-2 [26, 35].

In this study, Dll4 was amplified for the first time in the tumor endothelium, its predominant site of expression, and the consequences were analyzed in both grafted and in more representative autochthonous tumor models that better reflect host-tumor interaction and wherein the lesions arise and develop resembling the human disease. We consistently found that endothelial Dll4 overexpression reduces the growth of Lewis Lung Carcinoma (LLC) grafts, chemically-induced murine skin tumors as well as transgenic RIP1-Tag2 (RT2) mouse insulinomas, due to decreased vascular proliferation by modifying the activity of angiogenesis regulators. Importantly, we show that Dll4 overexpression reduces vascular responsiveness to VEGF seeming indicated for concomitant application with VEGF-inhibitors and stabilizes tumor circulation allowing for more efficient chemotherapy delivery while at the same time reducing the formation of distant-site metastasis.

## Methods

### Experimental animals

Double heterozygous Tie2-rtTA-M2 TetO7-Dll4 mice were generated as described [36] and used as hosts for tumor xenografts and chemically induced skin tumors. The RIP1-Tag2 (RT2) mice were kindly provided by Dr. Oriol Casanovas (Catalan Institute of Oncology) and used for breeding with Tie2-rtTA-M2 TetO7-Dll4 line for generation of triple mutants (RT2 Tie2-rtTA-M2 TetO7-Dll4) capable of developing pancreatic insulinomas and overexpress endothelial Dll4 after tetracycline or doxycycline-induction.

Restricted to the endothelium by the Tie-2 promoter, Dll4 overexpression was activated in inducible Tie2-rtTA-M2 TetO7-Dll4 (xenograft and skin tumorigenesis experiments) and RT2 Tie2-rtTA-M2 TetO7-Dll4 mutants (autochthonous insulinoma experiment) by administration *per os* of the tetracycline analogue doxycycline (2 mg/ml in drinking water *ad libitum*; Dll4 over-expression mice, D4OE). Non-induced Tie2-rtTA-M2 TetO7-Dll4 or RT2 Tie2-rtTA-M2 TetO7-Dll4 littermates, receiving just water, served as Dll4 basic expression controls (D4BE). Non-induced TetO7-Dll4 littermates, receiving 2 mg/ml doxycycline in drinking water ad libitum, served as Doxycycline control mice (D4BE + Doxy).

The animals were housed in ventilated propylene cages with sawdust bedding, in room with temperature between 22 °C and 25 °C and a 12-hours-light/12-hours-dark cycle. The mice were fed standard laboratory diet. From 12 weeks of age, RT2 and RT2 Tie2- rtTA TetO7-Dll4 mice received 5% sugar in drinking water to relieve hypoglycemia. All animal-involving procedures of this study were approved by the Faculty of Veterinary Medicine of Lisbon Ethics and Animal Welfare Committee (Approval ID PTDC/CVT71084/2012).

#### Xenograft mouse model

Male, 8-week old Tie2-rtTA TetO7-Dll4 mice (*n* = 12) were distributed into two equal groups that respectively continued receiving water (control Dll4-basic expression group, D4BE) or started receiving 2 mg/ml doxycycline in drinking water (Dll4 over-expression group, D4OE). A week later, Lewis lung carcinoma cells (LLC - ATCC® CRL- 1642^TM^, 10^6^) were implanted subcutaneously into the flank of each mouse. Injected sites were monitored daily. Once palpable, tumor largest (a) and smallest (b) diameters were measured and tumor volumes were calculated using the formula: V =  a × b^2^ × 0.52. Two weeks after the LLC injection, when the largest tumors approached the prescribed maximal, xenografts were dissected and processed for histological studies. For the metastasization experiment the basic protocol described above was used but the experiment was run for 6 weeks after the LLC injection.

#### Chemically-induced skin tumorigenesis model

Male, 8-week old Tie2-rtTA TetO7-Dll4 mice (*n* = 12) were separated into two equal groups and continued receiving respectively water (control *Dll4* basic expression group, D4BE) or started receiving 2 mg/ml doxycycline in drinking water (*Dll4* overexpression group, D4OE). A week later, all mice were shaved and treated with a single dose of 25 μg of 7,12-dimethylbenz[a]anthracene (DMBA; Sigma, St. Luis, MO) in 200 μL acetone per mouse applied to the dorsal skin. Beginning a week after DMBA-induction, tumor onset and growth was promoted by treating mice twice a week for 19 weeks with 4 μg of 12-O-tetradecanoylphorbol-13-acetate (TPA; Sigma, St. Luis, MO) in 100 μL of dimethyl sulfoxide (DMSO) per mouse. The appearance of skin lesion was monitored and recorded weekly. Mouse weight and tumor sizes (diameters) were periodically measured and lesion diameters were converted to tumor volume using the following formula: V = length × width × height × 0.52. Tumor burden of each individual mouse was calculated as the sum of individual tumor volumes. Twenty weeks after the DMBA-initiation, mice were euthanized and skin tumors were dissected and processed for histological and molecular analyses.

#### RIP1-*Tag2* (RT2) insulinoma model

RT2 Tie2-rtTA TetO7-Dll4 mice were generated as described above. Approaching the RT2 tumor stage conventionally used for therapeutic intervention assessments, 9.5-week old RT2 Tie2-rtTA TetO7-Dll4 mice (*n* = 16) were distributed into two equal groups that respectively started receiving water (control RT2 *Dll4* basic expression group, RT2 D4BE) or 2 mg/ml doxycycline in drinking water (RT2 *Dll4* overexpression group, RT2 D4OE). The mice were sacrificed at the age of 13.5 weeks just before most RT2 progenitors die due to tumor burden and hypoglicemia. The pancreata were dissected and macroscopic tumors (≥1 × 1 mm) were excised. Tumor volumes were calculated using the formula: V = length × width × height × 0.52. The volumes of all tumors from each mouse were added to give the overall tumor burden per animal. Subsequently, insulinoma samples were processed for histological analyses.

### Tumor tissue preparation, histopathology and immunohistochemistry

When dissected and measured as described above, tumors (LLC xenografts, skin lesions and RT2 insulinomas developed in D4BE and D4OE mice) were fixed in 4% paraformaldehyde (PFA) solution at 4 °C for 1 h, cryoprotected in 15% sucrose, embedded in 7.5% gelatin, frozen in liquid nitrogen and cryosectioned at 20 μm. For the metastasization experiment, lungs were dissected and macro-metastasis were observed under a dissection microscope. The lungs were resected for fixation in Bouin’s fixative for posterior metastasis histological confirmation. Skin tumor and insulinoma tissue sections were stained with Hematoxylin and Eosin (H&E) and subjected to review by a pathologist. Simultaneously, double fluorescent immunostaining to platelet endothelial cell adhesion molecule (PECAM) and pericyte marker alpha smooth muscle actin (α-SMA) was performed on xenograft and autochthonous skin and pancreatic tumor sections to examine tumor vascular density and vessel maturity. Rat monoclonal anti-mouse PECAM (BD Pharmingen, San Jose, CA) and rabbit polyclonal anti-mouse α-SMA (Abcam, Cambridge, UK) were used as primary antibodies and species-specific conjugated with Alexa Fluor 488 and 555 (Invitrogen, Carlsbad, CA) were used as secondary antibodies. Tissue sections were incubated with primary antibody overnight at 4 °C and appropriate secondary antibody for 1 h at room temperature. Nuclei were counterstained with 4´,6-diamidino-2-phenylindole dihydrochloride hydrate (DAPI; Molecular Probes, Eugene, OR). Stained sections were examined under a Leica DMRA2 fluorescence microscope with Leica HC PL Fluotar 10 and 20X/0.5 NA dry objective, captured using Photometrics CoolSNAP HQ,(Photometrics, Friedland, Denmark), and processed with Metamorph 4.6-5 (Molecular Devices, Sunnyvale, CA). The NIH ImageJ 1.37v program was used for morphometric analyses. Vessel density was estimated as the percentage of each tumor section field occupied by a PECAM-positive signal. Mural cell recruitment was assessed by quantitating the percentage of PECAM-positive structures lined by α-SMA-positive coverage.

### Perfusion study

To visualize the blood-perfused, i.e. functional, portion of the tumor circulation, mice were anesthetized and biotin-conjugated lectin from Lycopersicon esculentum (100 μg in 100 μl of PBS; Sigma, St. Luis, MO) was injected via caudal vein and allowed to circulate for 5 min before the animal vasculature was perfused transcardially with 4% PFA in PBS for 3 min. Tumor samples were collected and processed as presented above.

Tissue sections (20 μm) were stained with rat monoclonal anti-mouse PECAM antibody (BD Pharmingen, San Jose, CA), followed by Alexa 555 goat anti-rat IgG (Invitrogen, Carlsbad, CA). Biotinylated lectin was visualized with Strepatavidin-Alexa 488 (Invitrogen, Carlsbad, CA). The images were obtained and processed as described above. Tumor perfusion was quantified by determining the percentage of PECAM-positive structures that were co-localized with Alexa 488 signals corresponding to lectin-perfused vessels. In all cases, signal positive areas per microscopic field (*n* = 20 per mouse sample) were determined by the percentage of black pixels per field after transforming the RGB images into binary files.

### Quantitative transcriptional analysis

Using a SuperScript III FirstStrand Synthesis Supermix qRTPCR (Invitrogen, Carlsbad, CA), first-strand cDNA was synthesized from total RNA previously isolated with RNeasy Mini Kit (Qiagen, Valencia, CA) from skin tumors developed in D4BE and D4OE mice (*n* = 10 for each group). Real-time PCR analysis was performed as described (Trindade, Kumar et al. [37]) using specific primers for *β-actin*, *Pecam*, *Dll4*, *Hey2*, *Vegf-a*, *Vegfr1*, *Vegfr2*, *Vegf-c*, *Vegfr3*, *Pdgf-β* **,** *EphrinB2* and *Tie2*. Gene expression was normalized to *β-actin*. Primer pair sequences are available upon request.

### Total tumor doxorubicin quantitation

Total tumor doxorubicin was quantified using a method similar to Mayer et al [37]. At endpoint, 10% w/v tumor homogenates were prepared in tissue lysis buffer. Samples of the homogenate (200 uL) were placed in 2-mL microcentrifuge tubes, and 100 uL of 10% (v/v) Triton X-100, 200 uL of water, and 1500 uL of acidified isopropanol (0.75 N HCl) were added. After vortexing, doxorubicin was extracted overnight at -20 °C. The next day, the tubes were first vortexed and then centrifuged at 15,000 g for 20 min, and stored at -80 °C until analysis. Doxorubicin was quantified fluorometrically (kex = 470 nm, kem = 590 nm). To correct for nonspecific background fluorescence, the samples were compared with a standard curve made from the fluorescent emission of known amounts of doxorubicin added to acidified isopropanol extracts of homogenized tumor tissue from untreated mice. The data are expressed as microequivalents of doxorubicin/g tissue.

### Statistical analyses

Data processing was carried out by engaging Statistical Package for the Social Sciences version 15.0 (SPSS v. 15.0; Chicago, IL). Statistical analyses were performed using Mann-Whitney-Wilcoxon test. The results are presented as mean ± SEM or mean ± SD when more appropriate. *P*-values < 0.05 and <0.01 were considered significant (indicated in the figures with *) and highly significant (indicated with **), respectively.

## Results

### Endothelial Dll4 overexpression retards xenograft growth and reduces vascular response in LLC-bearing mice

**Fig. 1:**
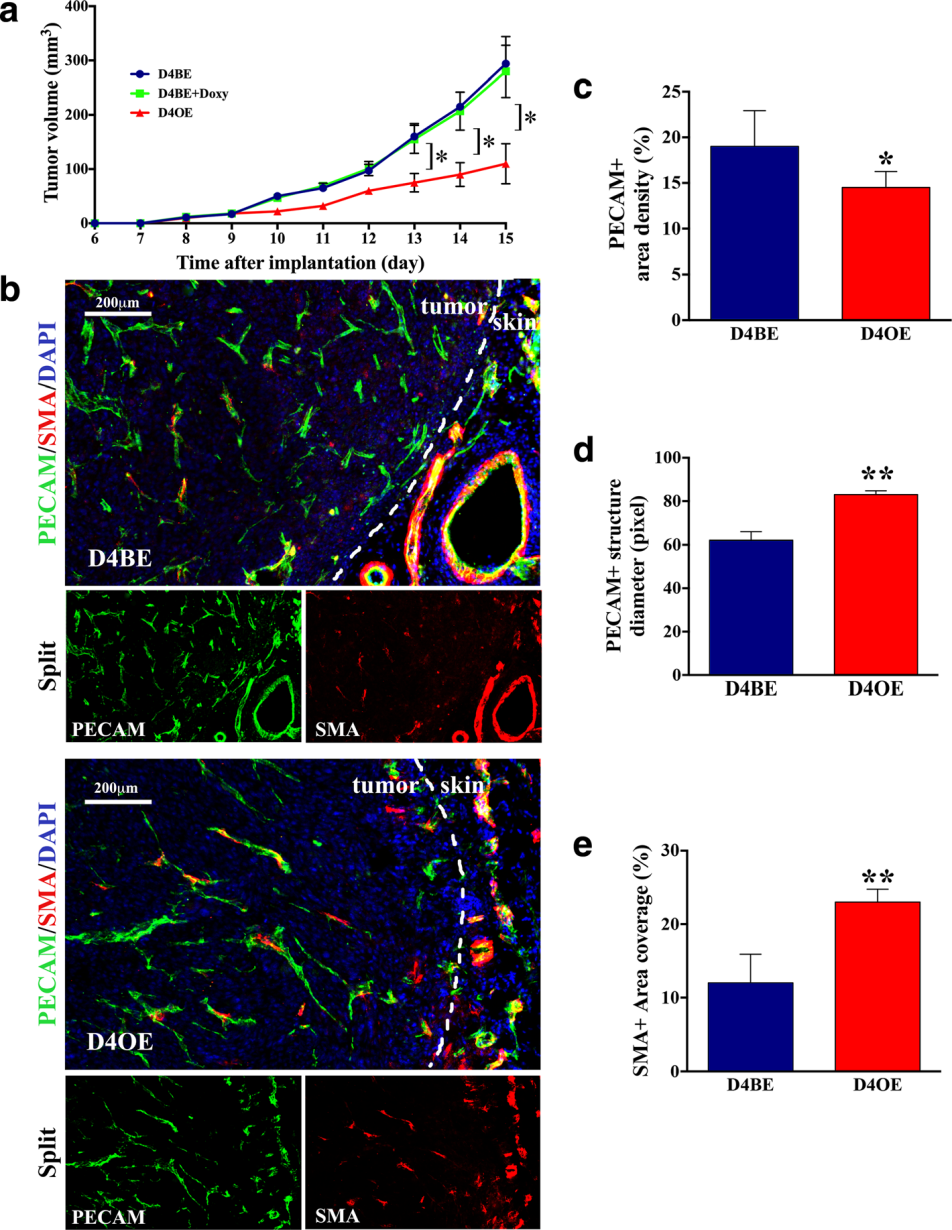
Endothelial Dll4 over-expression affects LLC subcutaneous xenograft growth and vasculature. **a** Dll4 over-expressed in endothelial cells retarded xenograft volume increase after subcutaneous LLC implantation in D4BE, D4BE + Doxy and D4OE littermates. *Error bars* represent SEM. *, *P* < 0.05 was considered significant. **b** Vascular response was examined in D4BE and D4OE tumor grafts using double immunostaining to endothelial PECAM and mural cell marker α-SMA. In relation with D4BE controls *(above*), the LLC xenografts developed in D4OE mice presented reduced vascular density (**c**), but increased vessel calibers (**d**) and promoted vessel wall assembly (**e**). Contrary to the D4BE tumor vessels that showed weak SMA staining and its co-localization with PECAM signals*,* SMA-positive cells lining PECAM-positive endothelium were distinctively increased in D4OE xenografts indicating improved vessel wall maturation. *Dot lines* mark tumor borders. Results are representative of *n* = 6 per mice group

To study the effects of endothelial Dll4 overexpression on tumor growth, LLC cells were injected subcutaneously in D4BE (non-induced Tie2-rtTA TetO7-Dll4), in D4BE + Doxy (doxycycline-induced TetO7-Dll4, which served as induction controls) and D4OE (doxycycline-induced Tie2-rtTA TetO7-Dll4) mice (*n* = 6). D4OE mice have been previously reported to efficiently overexpress Dll4 in the mouse embryonic [36] and adult vasculature [38], producing angiogenic arrest in active angiogenic areas while not affecting quiescent vasculatures [38]. The injected sites were monitored for a period of 15 days. In all mouse groups, the LLC tumors became palpable by day 8. D4BE + Doxy mice xenografts always grew at a similar rate to D4BE mice xenografts and were not further analyzed. However, D4OE mice revealed retarded xenograft expansion relative to D4BE mice throughout the experiment and displayed significantly smaller average tumor volumes beginning by day 13 (Fig. 1a). At the end of the experiment, all xenografts were removed and observed as encapsulated lesions while animals were inspected macroscopically and adjacent tissues as well as distant organs were found to be free of metastasis.

Subsequently, D4BE and D4OE tumors were analyzed for vascular morphologies by double immunostaining to endothelial marker PECAM and mural cell marker α-SMA. As presented in Fig. 1b, control D4BE tumors were found to be vascularized structures which fine and highly branched PECAM-positive (endothelial) networks lacked organized architecture and distinct hierarchy. Besides, newly forming vasculature showed weak α-SMA-positive mural cell recruitment, indicating low maturation rate. In comparison, D4OE tumors showed a reduced vascular response with decreased average PECAM-positive area density compared to D4BE xenografts (1.5-fold decrease, *p* < 0.05; Fig. 1c). Their vessels were found to be straighter and less branched, presenting, at the same time, wider calibers (Fig. 1d) and an highly significant increase in SMA-positive cell coverage relative to the control tumor vessels (>2-fold increase, *p* < 0.01; Fig. 1e). Thus, endothelial Dll4 overexpression, that caused the LLC implant growth retardation, was associated with reduced density of the endothelial network but promoted vascular stabilization as indicated by increased lumen diameters and vessel wall maturity.

### Dll4 overexpression inhibits the skin papilloma formation, restricts vessel proliferation and improves vascular functionality

**Fig. 2:**
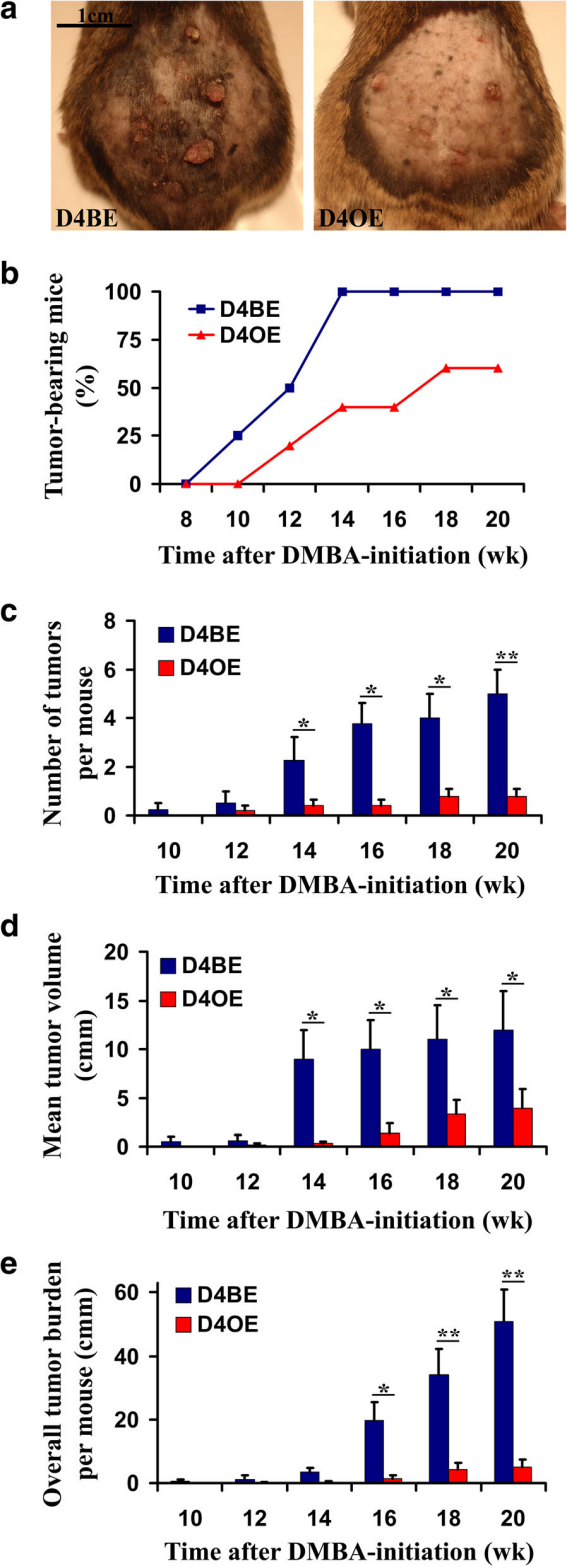
Endothelial Dll4 over-expression suppresses chemically- induced skin tumor onset and development. D4BE and D4OE mice were treated topically with a single dose of DMBA followed by two weekly applications of TPA for 19 weeks. **a** Representative pictures of D4BE and D4OE mice taken at the experiment end- point, i.e. 20 weeks after the DMBA- initiation, illustrate tumor-suppressing effect of endothelial Dll4 over-expression. **b** Percentage of tumor-bearing mice, number of tumors per mouse (**c**), mean tumor volume (**d**) and over-all tumor burden per mouse (**e**), calculated as the sum of tumor volumes developed by a mouse, throughout the tumor TPA-promotion in D4BE *vs.* D4OE mice. *Error bars* represent SEM. *, *P* < 0.05 and **, *P* < 0.01 were considered statistically significant and highly significant, respectively. Results are representative of *n* = 10 per mice group

To confirm the beneficial effects of endothelial Dll4 overexpression in an autochthonous tumor model, and to gain a more thorough insight into the morphological, functional and molecular consequences of increased Dll4/Notch signaling, skin tumorigenesis was initiated in D4BE and D4OE mice (*n* = 10) by a single topical DMBA treatment and then promoted by topical TPA applications twice per week for 19 weeks. As presented in Fig. 2a, b, D4BE mice started developing skin tumors as early as week 10 after DMBA-initiation and by week 12 of the study, 50% of D4BE mice developed at least one lesion (tumor latency). Tumor onset was delayed in D4OE animals for 2 weeks while tumor latency was extended up to week 17 of DMBA/TPA treatment. Beginning at week 14, we observed a statistically significant difference in the number of lesions per mouse (tumor multiplicity, Fig. 2c) and in the mean tumor volume between two experimental groups (*p* < 0.05; Fig. 2d). At the experiment end-point (20 weeks after DMBA-initiation), both tumor multiplicity and overall tumor burden, calculated as the sum of individual tumor volumes per mouse, were reduced with highly significant statistical difference (*p* < 0.01; Fig. 2e) in D4OE relative to control D4BE mice. Body weight of each mouse used in this study did not differ significantly prior to the start of, or at any time during the course of the experiment, suggesting that the level of carcinogen dosing was not toxic to the animals.

Subsequent histopathological analysis did not reveal any marked difference between D4BE and D4OE tumors. All lesions developed by D4BE as well as D4OE mice were exophytic lesions classified as benign squamous papillomas displaying hyperkeratotic epidermal projections with foci of dyskeratosis and dysplasia, intact basement membrane and superficial dermal inflammation.

**Fig. 3:**
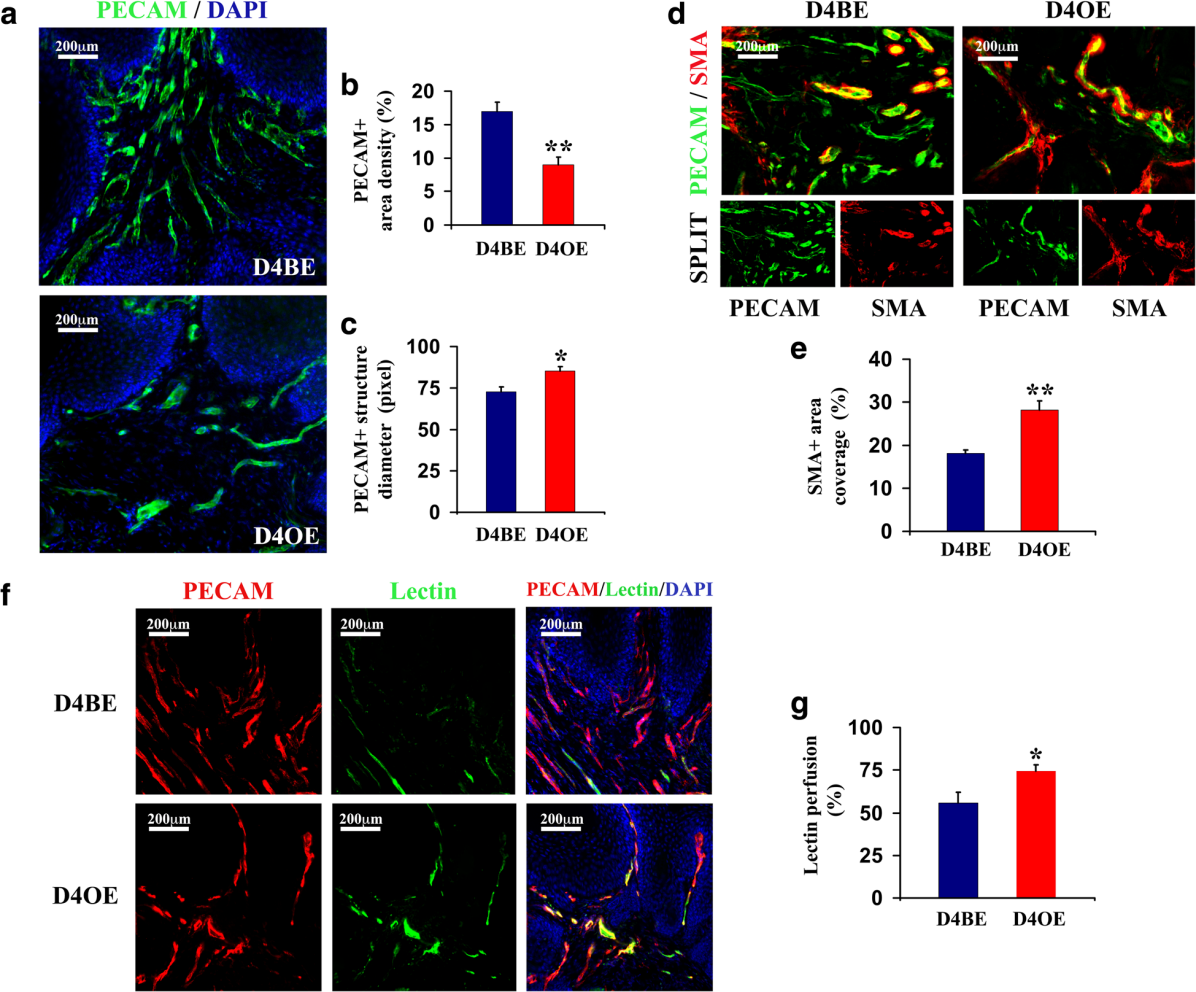
Vascular response upon skin chemical tumorigenesis in D4BE and D4OE mice. **a** Tumor endothelium was visualized by section PECAM immunostaining of skin tumors collected from DMBA/TPA-treated mice 20 weeks after the DMBA-initiation. Endothelial Dll4 over-expression was found to reduce endothelial system density by reducing its ramification (**b**), although branch diameters were observed increased in D4OE *vs.* D4BE mice (**c**). **d** Mural cell recruitment was assessed by PECAM/SMA co-immunostaining (a*bove*). **e** Endothelial Dll4 over-expression promoted vessel wall assembly which contributed to the normalization of tumor vasculature. **f** To evaluate tumor vessel competence, sub-groups of both D4BE and D4OE mice were perfused before being sacrificed with i.v. injected lectin as presented in “Materials and Methods”. Simultaneous immunostaining to PECAM and biotinylated lectin visualization with Streptavidin-Alexa 488 demonstrate increased fraction of competent vessels in D4OE *vs.* D4BE skin tumors (**f**). Percentage of PECAM-positive area co-localized with lectin (Alexa 488 signal) was measured to quantify the portion of functional vessels within skin tumors (**g**). The images presented in panels **a**-**g** were captured and processed as described in Fig. 1. *Error bars* represent SEM. *, *P* < 0.05 and **, *P* < 0.01 were considered statistically significant and highly significant, respectively. Results are representative of *n* = 10 per mice group

Immunostaining to PECAM indicated highly significant microvascular density reduction (*p* < 0.01) in papillomas of D4OE vs. D4BE mice (Fig. 3a,b). Comparable to morphological changes observed in subcutaneous xenografts, Dll4 overexpression was shown to restrict PECAM-network branching but increase papilloma vessel lumen calibers (*p* < 0.05, Fig. 3c), α-SMA-positive mural cell recruitment (*p* < 0.01, Fig. 3d,e) and promote vessel competence as indicated by significantly increased lectin perfusion in papillomas developed by D4OE vs. D4BE (*p* < 0.05, Fig. 3f,g). In this way, retarded growth of autochthonous skin tumors under the condition of increased endothelial Dll4/Notch signaling was demonstrated to be a consequence of reduced tumor vascular density, even though Dll4 over-expression was simultaneously found to augment individual papilloma vessel functional capacity in papillomas.

### Increased Dll4/Notch signaling affects the expression of principal angiogenesis regulators in chemically-induced skin papillomas

**Fig. 4:**
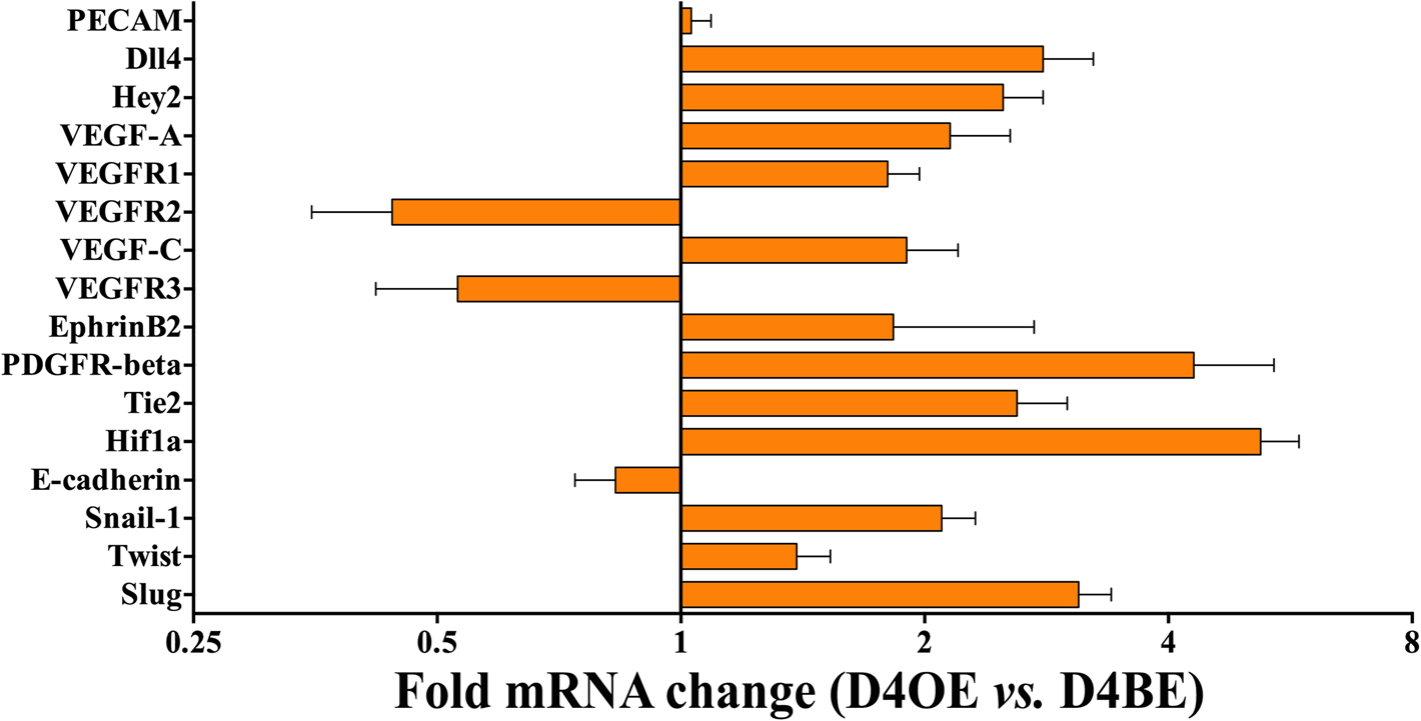
Differential gene activity between chemically-induced skin tumors of D4BE and D4OE mice. Expressions of selected genes in tumor tissue samples were examined using quantitative RT-PCR. Tests were done in triplicate. All RT-PCR data were normalized to β-actin levels. For each gene, relative mRNA levels of D4OE tumor samples were compared to the reference D4BE levels and presented as x-fold change. *Error bars* represent SEM

To study the molecular changes underlining morphological and functional vessel alterations caused by increased Dll4 expression, we used qRTPCR and analyzed D4BE and D4OE papillomas for differential expression of the main genes implicated in angiogenesis regulation (Fig. 4.). Compared to D4BE papillomas, D4OE tumors had on average 2.8-fold increased *Dll4* mRNA levels and 2.5-fold up-regulated *Hey2* transcription confirming the Dll4/Notch pathway over-induction. The *Vegf-a* mRNA levels were augmented probably due to increased hypoxia; nevertheless, dramatically reduced sensitivity to this major pro-angiogenic factor was indirectly indicated by a ~2-fold increase of the trapper-receptor *Vegfr1* transcription and a >2-fold reduction of the main signaling receptor *Vegfr2* transcription. Similarly, Vegf-c was up-regulated while the transcription of its receptor *Vegfr3* was shown to be reduced. As expected, our analysis demonstrated an increase of *Pdgfr-β*, *EphrinB2* and *Tie2* activity accompanying increased mural cell recruitment and vascular stability. To evaluate the impact of endothelial Dll4 overexpression on epithelial homeostasis we also evaluated the transcriptional changes of *E-cadherin*, as an epithelial marker, *Snail-1*, *Twist* and *Slug* as Epithelial-to-mesenchymal transition (EMT) markers and *Hif1-a* as a hypoxia marker. The results demonstrated that D4OE mice tumors display increased EMT markers and *Hif1-a* transcription.

### Endothelial Dll4 overexpression reduces RT2 insulinoma growth, decreases vascular density and stabilizes tumor circulation

**Fig. 5:**
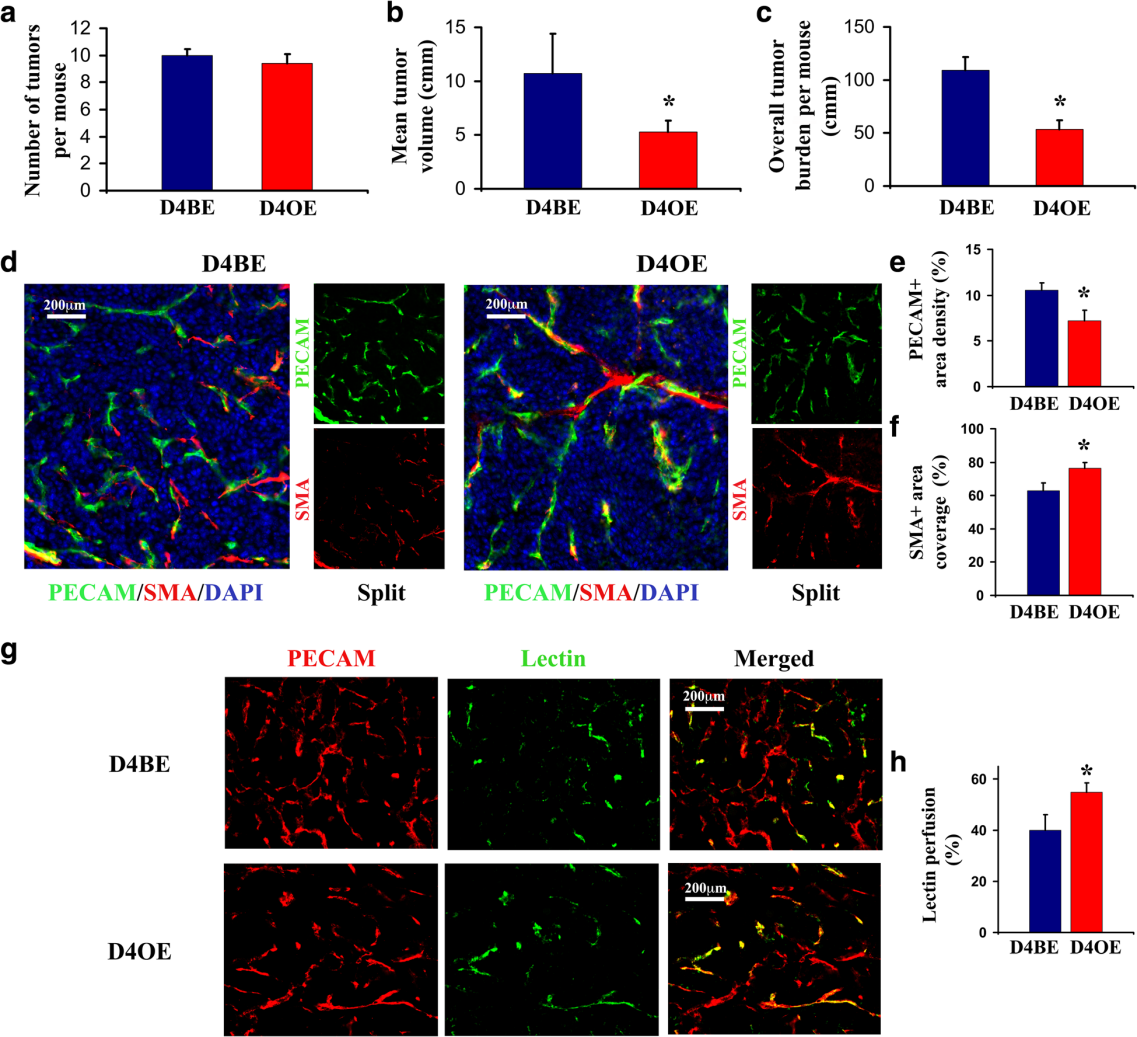
The endothelial Dll4 over expression effects on RT2 insulinoma kinetics and vascular response. Dll4 over-expression in endothelial cells was induced in 9.5-week old RT2 Tie2-rtTA TetO7-Dll4 mice, maintained for 4 weeks by doxycycline p.o. application and assessed as a potential therapeutic strategy in comparison with non-induced RT2 Tie2-rtTA TetO7-Dll4 littermates. Increased endothelial Dll4 expression was demonstrated to be a powerful tumor-suppressor intervention in RT2 mice recapitulating the vascular phenotype previously observed in grafted and autochthonous skin tumors. **a** Number of tumors per mouse, mean tumor volume (**b**) and over-all tumor burden per mouse (**c**), calculated as the sum of tumor volumes developed by a mouse, in 13.5-week old RT2 D4BE *vs.* RT2 D4OE mice. **d** Double immunostaining to PECAM/SMA indicating reduced microvessel density (**e**) and increased mural cell recruitment (**f**) in RT2 D4OE *vs*. RT2 D4BE insulinomas (*left*). **g** Vascular functionality was examined by mouse lectin perfusion. Simultaneous immunostaining to PECAM and biotinylated lectin visualization with Streptavidin-Alexa 488 demonstrate increased portion of perfused vessels in RT2 D4OE *vs.* RT2 D4BE tumors (**h**). Percentage of PECAM-positive area co-localized with lectin signals was measured to quantify the portion of competent vessels within pancreatic tumors (**h**). The images presented in panels *D* and *G* were captured and processed as described in Fig. 1. *Error bars* represent SEM. *, *P* < 0.05 was considered statistically significant. Results are representative of *n* = 10 per mice group

To confirm that Dll4 overexpression results in tumor suppression independently of neoplasm histological type, we also studied its impact on autochthonous RT2 insulinoma development. In this model, pancreatic islet carcinogenesis develops as a result of the simian virus SV-40 large T-antigen expression that is restricted to the insulin-producing β cells of the Langerhans islets due to the control of the Rat Insulin Promoter [39]. Although oncogene expression is detectable as early as embryonic day E8.5, RT2 mice are born with normal pancreatic histology and hyperplastic/dysplastic islets begin to appear later, at 4-5 weeks of age, progressing during the next 5 weeks into angiogenic islets, subsequently encapsulated adenomas (insulinomas) and finally invasive carcinomas [40]. We generated RT2 Tie2-rtTA-M2 TetO7-Dll4 mice and simulated a therapeutic intervention by inducing endothelial Dll4 overexpression in a mouse group (RT2 D4OE) vs. non-induced control group (RT2 D4BE) (*n* = 10) during the developing RT2 tumor stage (10 – 13.5 weeks old animals). When dissected in the phase corresponding to RT2 terminal-disease, RT2 D4BE and RT2 D4OE mice were found not to differ in terms of average number of insulinomas per individual (Fig. 5a). However, average tumor volume (Fig. 5b) and overall tumor burden were significantly reduced in RT2 D4OE compared to RT2 D4BE mice (~50% reduction, *p* < 0.05; Fig. 5c). Histologicaly, Dll4 overexpression in RT2 D4OE mice was found to decrease insulinoma endothelial density by 32% (*p* < 0.05; Fig. 5d,e), increase α-SMA-positive mural cell coverage by ~20% (*p* < 0.05; Fig. 5d,f) and result in improved lectin perfusion by 27% (*p* < 0.05) in relation with RT2 D4BE insulinomas (Fig. 5g,h). Thus, amplified Dll4/Notch signaling repeated its effects on tumor growth and modified vascular response in RT2 insulinomas in the same way it previously influenced angiogenesis in LLC xenografts and chemically-induced skin tumors.

### Endothelial Dll4 overexpression reduces metastasis formation and improves accumulation of concomitantly administrated chemotherapy in primary tumor

**Fig. 6:**
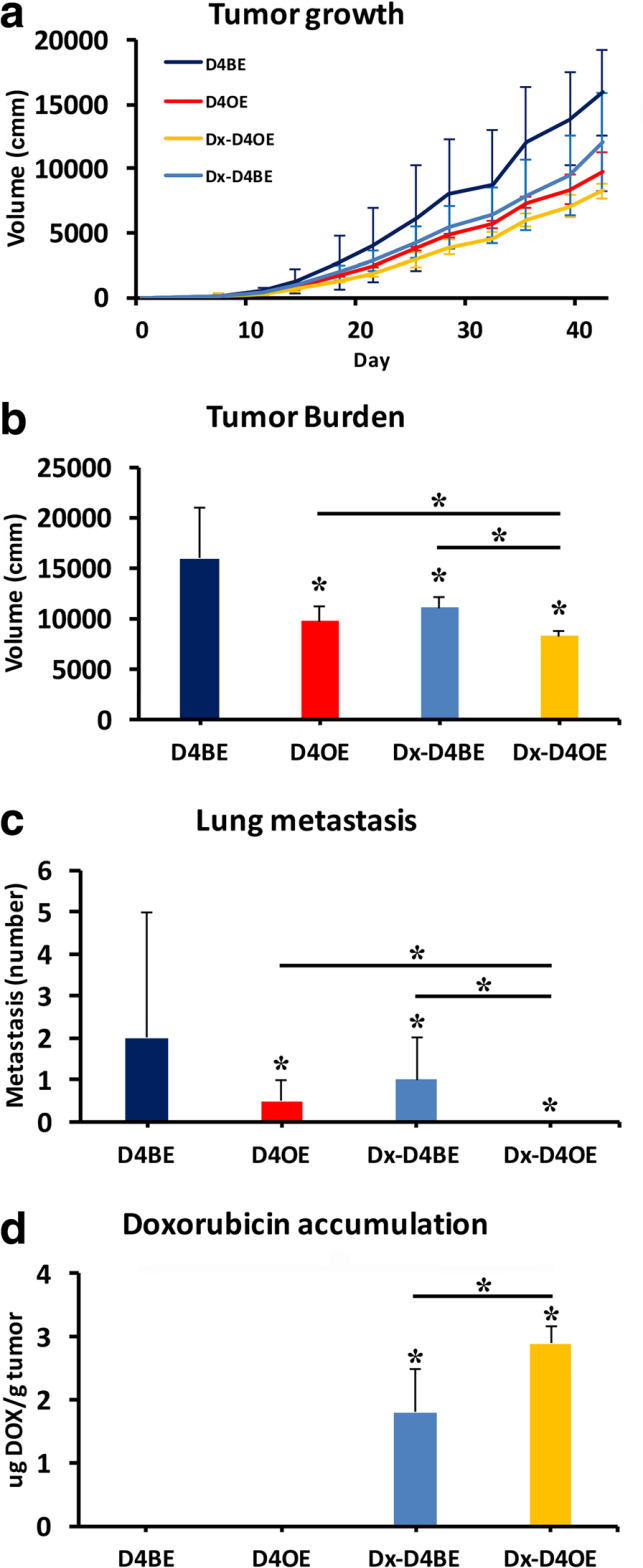
Endothelial Dll4 over expression improves tumor drug delivery and reduces metastasis formation. **a**, **b** doxorubicin administration (Dx-D4BE) and Dll4 over-expression in endothelial cells (D4OE) retarded xenograft volume increase after subcutaneous LLC implantation in D4BE and D4OE littermates. Doxorubicin administration to Dll4 endothelial overexpressing mice (Dx-D4OE) resulted in the highest tumor growth regression. Upon endpoint lungs were dissected and metastatic foci were counted under a dissection microscope. Both D4OE and Dx-D4BE mice displayed reduced numbers of lung metastasis but Dx-D4OE mice had no detectable lung metastatic foci (**c**). **d** Primary tumor samples were also recovered at endpoint to evaluate doxorubicin concentration. Doxorubicin accumulation in the primary tumor was increased by 60% in D4OE mice when compared to D4BE. *Error bars* represent SEM. *, *P* < 0.05was considered significant. Results are representative of *n* = 10 per mice group

To test if the vascular normalization induced by Dll4 overexpression had an impact on tumor drug delivery and the formation of distant site metastasis, we used a subcutaneous Lewis Lung Carcinoma (LLC) tumour transplant mouse model. Mice were palpated daily until detection of first solid masses was possible, which happened at day 7. At day 8, we started administrating doxycycline in the drinking water to test mice, separating Dll4BE and Dll4OE test groups (*n* = 10), and tumor volume was measured until the end-point (6 weeks after LLC cells injection). At day 11 we started administrating doxorubicin (2 mg/kg, 3x a week) to Dll4BE and Dll4OE mice separating further two groups Dx-Dll4BE and Dx-Dll4OE (*n* = 5). Doxorubicin is a member of the anthraciclines family of chemotherapeutic drugs. As shown in Fig. 6a, tumor growth was reduced by both endothelial Dll4 overexpression and doxorubicin administration to Dll4BE mice, while the concomitant administration of doxorubicin to Dll4OE mice further reduced the tumor growth by 15% at endpoint (*p* < 0.05; Fig. 6b). Lung metastasis were counted at endpoint. While Dll4OE mice displayed a 4x reduced number of metastatic foci when compared to control mice, no metastatic foci were detected in Dll4OE mice administered with doxorubicin (*p* < 0.05; Fig. 6c). Evaluation of doxorubicin concentration in the primary tumor revealed that endothelial Dll4 overexpression improved drug accumulation by 60% (*p* < 0.01; Fig. 6d).

## Discussion

The inhibition of Dll4/Notch signaling was demonstrated in preclinical models to induce immature and non-functional vessel proliferation on an accelerated rate and result in poor blood supply and consistent growth inhibition of different mouse, rat and human tumors [15–17, 25]. Nevertheless, Dll4/Notch signaling-blockade remains a strategy with unpredictable clinical usefulness. Basically, vascular defects self-repair and reperfusion over long-term Dll4/Notch-suppression may revert tumor growth, particularly in association with increasing malignant cell invasiveness, previously documented as a consequence of antiangiogenic-induced hypoxia [29]. Besides, reduced vessel competence due to Dll4/Notch-inhibition can be expected to limit concomitant chemotherapy effectiveness. Therefore, we examined the effects of the opposite strategy, based on endothelial Dll4 overexpression, which was anticipated to reduce vascular response within tumors and suppress their expansion. In addition, Dll4 overexpression was expected to promote vessel maturation and stabilize the tumour vasculature by reducing its remodeling capacity and, in this way, the risk of development of therapy resistance and also improve tumor drug delivery.

Our results demonstrate that endothelial Dll4 overexpression reduces the growth of LLC xenografts, autochthonous chemically-induced skin papillomas and RT2 insulinomas. In all three models, remarkable tumor burden reduction due to Dll4 overexpression was consistently associated with decreased endothelial density and presumably reduced overall tumor blood supply. In contrast, vascular maturity and functionality were improved as evidenced by the formation of larger branches, increased vessel network perfusion and increased mural cell recruitment. However, improved vessel competence was not found to be predominant over the beneficial effects caused by restricted vessel proliferation but could result in better cytostatic or other drug delivery at the tumor site. In addition, the enhanced vessel wall maturation seen in endothelial Dll4 overexpressing mice may help to prevent the penetration of the malignant cells into the circulation and subsequent metastasization.

The comparative gene expression analysis of skin tumors developed by wild-type vs. Dll4 overexpression mice confirmed that Dll4/Notch signaling restricts VEGF dependent neoangiogenesis. Although *Vegf-a* was found to be up-regulated under the conditions of amplified Dll4/Notch signaling, which is likely to be due to increased hypoxia revealed by elevated *Hif1-a* transcription, reduced vascular sensitivity to VEGF-A was achieved by reduced *Vegfr2* transcription and simultaneous up-regulation of *Vegfr1*, which lacks significant signaling activity in endothelial cells. Explaining, at least partially, the molecular mechanisms leading to reduced vascular response in Dll4 overexpressing vs. control tumors, these findings also point out the potential capacity of endothelial Dll4 overexpression to increase the efficacy of currently available VEGF signaling-inhibitors whose clinical success has been limited by development of tumor-resistance. Similarly to *Vegf-a*, *Vegf-c*, a positive driver of normal lymphangiogenesis and an additional tumor pro-angiogenic factor [41], was also found up-regulated possibly due to more pronounced hypoxia while Dll4 overexpression in tumor endothelial cells decreased receptor Vegfr3 levels and, thereby, tumor vascular sensitivity to VEGF-C.

Concerning the improved perivascular cell recruitment, we found Dll4 overexpression to influence the transcription of both *Ephrin-B2* and platelet-derived growth factor receptor beta (*PDGFRβ*). In several developing tissues, binding of Ephrin-B2 to its receptor, EphB4,modulates adjacent endothelial cell interactions, while Ephrin-B2/EphB4 signaling between endothelial and mural cells controls mural cell motility and adhesion [15, 42]. In parallel, high levels of platelet-derived growth factor B (PDGFB) in proliferating endothelial cells promote the recruitment of pericytes that express the PDGFRβ [42]. Our evidence suggests that Dll4/Notch signaling amplification stabilizes tumor vessels by enhancing EphrinB2/EphB4 and PDGF/ PDGFRβ signaling and, therefore, promoting vascular maturation. Induced *Tie2* transcription can be considered complementary since Tie2, when activated by angiopoietin1, is essential to maintain the endothelium in the quiescent state [43] by promoting mural cell recruitment [44].

Epithelial homeostasis was also revealed to be changed from endothelial Dll4 overexpression. Epithelial marker *E-cadherin* transcription was downregulated while EMT markers *Snail-1*, *Twist* and *Slug* were all upregulated. This is indicative of a higher pressure towards the metastatic phenotype in D4OE mice. Both the potential benefit of Dll4 overexpression in increasing chemotherapy effectiveness and its influence on metastasis formation were also evaluated in this study by use of a metastasizing LLC xenograft protocol. We understand there are advantages to using an orthotopic mouse model of metastisation [22] instead of a xenograft protocol, especially because the tumor microenvironment is so different but the protocol we chose allowed us to explore the lung tropism of the LLC cells to more effectively restrict and direct circulating tumor cells to the lungs. Results revealed that endothelial Dll4 overexpression and the independent administration of doxorubicin, a common chemotherapeutic drug, were equally effective in reducing tumor burden and the formation of distant-site metastasis. It is also worth making note that results were always found to be tendentially better for the D4OE group but never significantly different from independent administration of doxorubicin. However, the administration of doxorubicin to D4OE mice resulted in the highest reduction of tumor growth and endpoint tumor burden, with no detectable metastasis found in the lungs of test mice. Evaluation of primary tumor drug accumulation revealed that doxorubicin accumulation was increased by 60% when endothelial cells were overexpressing Dll4. The highly significant decrease in metastasis formation in D4OE mice contrasts with the increase in EMT markers previously observed. This could be indicative that tumor cells that become malignant could be failing to effectively intravasate the highly smooth muscle cell covered neovasculature of the primary tumor and become trapped.

The results presented here represent the opposite to those described by Dll4/Notch genetic or pharmacologic inhibition when we look at the endothelial or smooth muscle cell layer phenotypes. Nevertheless, in both cases we observe a reduction of tumor growth. Probably because in both cases, despite opposing vascular phenotypes, tumor hypoxia is increased. Something similar was previously reported by us in a wound healing setting [38]. Also in that case, both endothelial Dll4 loss- and gain-of-function resulted in impaired wound regeneration despite having opposing vascular phenotypes. As in that case, tissue dynamics depend more on vascular function than morphology.

Transduced malignant cells that over-express membrane Dll4 (entire molecule or functional extra-cellular portion) were previously found in subcutaneous tumor grafts to result in reduced tumor vessel density and produce wide, straight and less branched vessels [17, 26, 34]. Although conflicting data were obtained regarding these neovessel functional capacities and repercussions on tumor expansion, a significant number of tumor lines responded with regression to Dll4 overexpression while quite minimal Notch activation was noted in several other tumors characterized as unresponsive to Dll4/Notch activation [34]. This study focused on endothelial Dll4 overexpression, since Dll4/Notch predominantly mediates EC-EC rather than malignant cell-EC communication, as simulated in previous Dll4 overexpression experiments, even though the Dll4 molecule does appear in a wide range of human malignant cells [22]. In addition, considering the accessibility of the tumor endothelium, therapeutic Dll4 delivery to the neoplastic cells seems much more complex to implement than endothelial targeting. More importantly, as the generalized Notch1/4 activation by a systemic agonist could produce several side-effects caused by the perturbation of different Notch-dependent physiological processes, selective *Dll4* genetic sequence delivery, e.g. using endothelial-specific liposomes, might restrict Notch over-activation to sites of active angiogenesis.

## Conclusions

In summary, this study suggests that endothelial Dll4 overexpression may constitute an effective mean to suppress tumor angiogenesis, neoplasm growth and metastasis formation, without observed toxic side effects. Mechanistically, it has potential to provide synergy with VEGF inhibitors and enhance chemotherapy effectiveness. Therefore, therapeutic endothelial Dll4 overexpression is worth further investigation.

## Abbreviations

cDNA: Complementary deoxyribonucleic acid
D4BE: Tie2-rtTA-M2 TetO7-Dll4 mice administered normal drinking water
D4OE: Tie2-rtTA-M2 TetO7-Dll4 mice administered 2 mg/ml doxycycline in drinking water
DAPI: 4´,6-diamidino-2-phenylindole dihydrochloride hydrate
Dll4: Delta-like 4
DMBA: 7,12-dimethylbenz[a]anthracene
DMSO: Dimethyl sulfoxide
E: Mouse embryonic day
Hes: Hairy and enhancer of split (Drosophila)
Hey: Hairy/enhancer-of-split related with YRPW motif 1
kem: Emission wavelength
kex: Excitation wavelength
LLC: Lewis lung carcinoma
NICD: Notch intracellular domain
PBS: Phosphate-buffered solution
PECAM: Platelet endothelial cell adhesion molecule
PFA: Paraformaldehyde
qRTPCR: Real-time quantitative reverse transcription PCR
RBP-Jk: Recombination signal binding protein Jk
RGB: Red green blue truecolor image
RIP1: Rat insulin promoter 1
RT2: RIP1-Tag2 mice
rtTA: Reverse tetracycline-controlled transactivator
SD: Standard deviation
SEM: Standard error of the mean
Tag2: Simian virus 40 large and small T-antigens
TetO7: Seven copies of the Tet repressor binding site
Tie2: Endothelial-specific receptor tyrosine kinase 2
TPA: 12-O-tetradecanoylphorbol-13-acetate
VEGF: Vascular endothelial growth factor
α-SMA: Alpha smooth muscle actin
